# Dynamic Softening and Hardening Behavior and the Micro-Mechanism of a TC31 High Temperature Titanium Alloy Sheet within Hot Deformation

**DOI:** 10.3390/ma14216515

**Published:** 2021-10-29

**Authors:** Kexin Dang, Kehuan Wang, Gang Liu

**Affiliations:** 1 National Key Laboratory for Precision Hot Processing of Metals, Harbin Institute of Technology, Harbin 150001, China; dangkexinhit@163.com (K.D.); gliu@hit.edu.cn (G.L.); 2 Institute of High Pressure Fluid Forming, Harbin Institute of Technology, Harbin 150001, China

**Keywords:** titanium alloy, dynamic softening, dynamic hardening, recrystallization

## Abstract

TC31 is a new type of α+β dual phase high temperature titanium alloy, which has a high specific strength and creep resistance at temperatures from 650 °C to 700 °C. It has become one of the competitive candidates for the skin and air inlet components of hypersonic aircraft. However, it is very difficult to obtain the best forming windows for TC31 and to form the corresponding complex thin-walled components. In this paper, high temperature tensile tests were carried out at temperatures ranging from 850 °C to 1000 °C and strain rates ranging from 0.001 s^−1^ to 0.1 s^−1^, and the microstructures before and after deformation were characterized by an optical microscope, scanning electron microscope, and electron back-scatter diffraction. The dynamic softening and hardening behaviors and the corresponding micro-mechanisms of a TC31 titanium alloy sheet within hot deformation were systematically studied. The effects of deformation temperature, strain rate, and strain on microstructure evolution were revealed. The results show that the dynamic softening and hardening of the material depended on the deformation temperature and strain rate, and changed dynamically with the strain. Obvious softening occurred during hot tensile deformation at a temperature of 850 °C and a strain rate of 0.001 s^−1^~0.1 s^−1^, which was mainly caused by void damage, deformation heat, and dynamic recrystallization. Quasi-steady flowing was observed when it was deformed at a temperature of 950 °C~1000 °C and a strain rate of 0.001 s^−1^~0.01 s^−1^ due to the relative balance between the dynamic softening and hardening. Dynamic hardening occurred slightly with a strain rate of 0.001 s^−1^. Mechanisms of dynamic recrystallization transformed from continuous dynamic recrystallization to discontinuous dynamic recrystallization with the increase in strain when it was deformed at a temperature of 950 °C and a strain rate of 0.01 s^−1^. The grain size also decreased gradually due to the dynamic recrystallization, which provided an optimal forming condition for manufacturing thin-walled components with the desired microstructure and an excellent performance.

## 1. Introduction

Titanium alloys are widely used in aerospace and other industrial fields due to their excellent properties, such as a high specific strength, corrosion resistance, and creep resistance [1,2,3]. In recent years, the demand for high-temperature titanium alloys has increased with the development of new generation aircraft aiming to be high-speed and lightweight [4,5]. The ultimate strength of the TC31 titanium alloy at 650 °C can reach more than 600 MPa [6,7]. Therefore, the TC31 titanium alloy meets the requirements of structural parts that are served short-time at 650 °C~700 °C such as the skins and air inlets of hypersonic aircrafts [2,5,6]. However, titanium alloys have high strength, poor ductility, and a low Young’s modulus at room temperature, so it is usually necessary to heat titanium alloys to a certain temperature for hot forming, such as hot forging, hot pressing, superplastic forming, and hot gas pressure forming, etc. [8,9,10,11]. Hot gas pressure forming is a process that can form titanium alloy complex thin-walled components with a relatively high efficiency. However it is very challenging to control the deformation uniformity during the hot gas pressure forming process of thin-walled components, which may give rise to local thinning and cracking defects [12,13,14,15]. The deformation uniformity is affected by the coupling of dynamic hardening and dynamic softening during the hot deformation. The material flow behavior is complex and very sensitive to thermomechanical factors such as temperature, strain rate, and strain [16,17]; therefore, it is very challenging to accurately control the post-form microstructure and properties of high-temperature titanium alloy thin-walled parts.

Lots of studies have been performed on the hot deformation behavior and microstructure evolution of near-α titanium alloys. Hao et al. [18] studied the deformation mechanisms of the Ti-6Al-2Zr-1Mo-1V titanium alloy in a wide temperature range from −60 °C to 900 °C and found that the deformation mechanism was dislocation slip when the deformation temperature was below 600 °C, but the dynamic recrystallization and the spheroidization of lath α resulted in the flow softening and significantly decreased the flow stress when the deformation temperature was above 600 °C [19]. During the superplastic tensile deformation of the Ti55 titanium alloy at 925 °C and 0.00664 s^−1^ [20], the main mechanism was grain rotation accommodated by grain boundary sliding, and the softening during the hot tensile tests was mainly attributed to DDRX. During the isothermal multi-directional forging of the Ti-6.5Al-2.5Sn-9Zr-0.5Mo-1Nb-1W-0.25Si titanium alloy at the temperature range of 920 °C~1010 °C [21], the recrystallization mechanism changed from discontinuous dynamic recrystallization (DDRX) to continuous dynamic recrystallization (CDRX), and the grain size became smaller with the decrease in the deformation temperature. Wu et al. [22] pointed out that the hot tensile deformation of the TA32 high temperature titanium alloy rolled sheet at 700 °C~900 °C was affected by anisotropy, and the main dislocation slip types were cylindrical slip and conical slip along the rolling direction and transverse direction, respectively, but DDRX could effectively weaken the anisotropy at a higher deformation temperature. The flow softening mechanisms of the IMI834 high temperature titanium alloy during the isothermal compression deformation in the α+β two-phase region and the β single-phase region were dynamic recrystallization (DRX) and dynamic recovery (DRV), respectively [23]. When the deformation occurred at the β single-phase region, the cross slip and climb of dislocations easily occurred. The research on the hot deformation mechanisms of dual-phase titanium alloys has mainly concentrated on the TC4 titanium alloy [24]. At present, there are few reports on the hot deformation behavior and microstructure evolution of the new developed TC31 dual-phase high temperature titanium alloy.

In this paper, the hot deformation behaviors of the TC31 titanium alloy at 850 °C~1000 °C and 0.001 s^−1^~0.1 s^−1^ were investigated firstly by uniaxial tensile tests, and the dynamic softening and hardening of the material were analyzed according to the obtained flow stress curves. Secondly, the microstructure evolution and mechanisms, especially the recrystallization behaviors, under different deformation temperatures, strain rates, and strains were systematically studied by scanning electron microscopy (SEM) and electron backscatter diffraction (EBSD). This work could provide some guidence for the forming of TC31 high temperature titanium alloy thin-walled components, and promote the industrial application of this newly developed titanium alloy.

## 2. Materials and Methods

### 2.1. Materials

**Figure 1:**
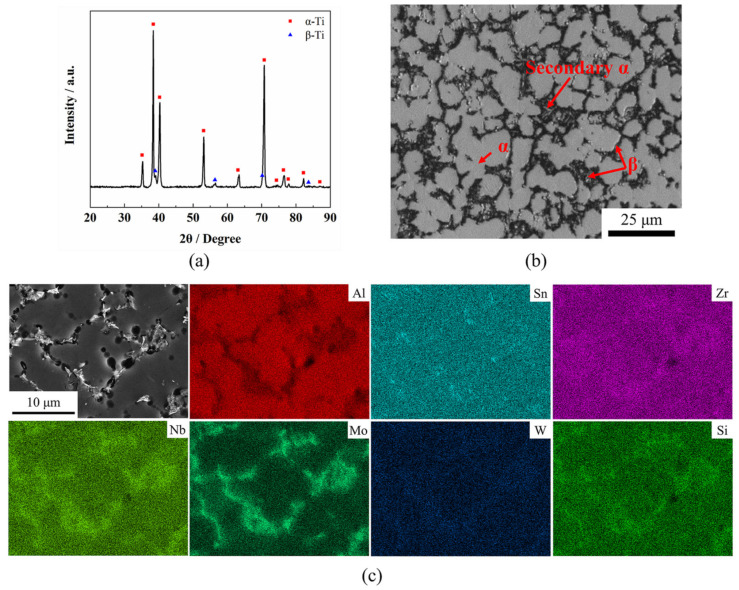
(**a**) The XRD of the TC31 titanium alloy; (**b**) microstructure of the as-received sheet; (**c**) the EDX elemental mapping of the TC31 titanium alloy.

The material used in this study was a TC31 high-temperature titanium alloy hot-rolled sheet with an average thickness of 1 mm, which was supplied by Baotai Group Co., Ltd. (Baoji, China). The chemical compositions obtained by PW4400 X-ray fluorescence spectrometer are listed in Table 1. The x-ray diffraction (XRD) analysis of the TC31 titanium alloy sheet was performed by an X’PERT PRO diffractometer (Malvern Panalytical Co. Ltd., Almelo, The Netherlands) and the results are shown in Figure 1a. The XRD patterns indicate that the TC31 titanium alloy consists of α phase and β phase. The initial microstructure of the hot-rolled sheet consists of a mostly equiaxed α phase, a few lamellar secondary α phase, and some strip and block shaped β phase. The β phase is distributed along the grain boundaries of the α phase, and the volume fraction of the β phase is about 20%, as shown in Figure 1b. The β transition temperature was confirmed to be 1025 °C by the metallographic method. The EDX elemental mapping of the TC31 titanium alloy is shown in Figure 1c. Regarding the TC31 titanium alloy, the α-stabilizing element includes Al, the β-stabilizing elements include Nb, Mo, W and Si, and the neutral elements include Sn and Zr [25]. It is clear that the distributions of neutral elements are uniform and the distributions of the remaining elements are non-uniform. The concentration of the Al element is higher in the α phase and the concentrations of Nb, Mo, W, Si elements are higher in the β phase, which is typical for titanium alloys. Nb and Mo are β isomorphous elements, so their concentration contrast in the α phase and the β phase is obvious; however, W and Si are β eutectoid elements and their concentration contrast in the α phase and the β phase are slight.

### 2.2. Uniaxial Tensile Tests and Microstructure Characterization

The selection of the parameters was based on the hot gas pressure forming process for the TC31 titanium alloy. The forming parameters should ensure not only the formability but also the post-form properties. Regrading the TC31 titanium alloy, the peak stress at 850 °C is as high as 250 MPa at the strain rate of 0.01 s^−1^, which is too high for the hot gas pressure forming process. Hence, the minimum tensile temperature was chosen as 850 °C. Titanium alloy thin-walled components are usually formed in the α+β two-phase zone in consideration of the post-form mechanical properties [2,9,13]. In this paper, the β transition temperature of the TC31 titanium alloy was confirmed to be 1025 °C by the metallographic method; therefore, the maximum tensile temperature was chosen as 1000 °C. The strain rate during the process of the hot gas pressure forming was about 0.01 s^−1^ [3,8,15]; therefore, the range of strain rate in this study was chosen from 0.001 s^−1^ to 0.1 s^−1^.

**Figure 2:**
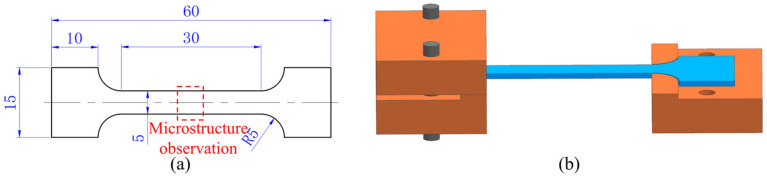
(**a**) Geometry dimensions of the tensile specimens (unit: mm); (**b**) schematic diagram for the clamping fixture.

Specimens for the high temperature tensile tests were machined by wire-cut electrical discharge machining from the as-received sheet with the tensile axis parallel to the rolling direction, and the dimensions are shown in Figure 2a. Firstly, the oxide layer of the TC31 titanium alloy was removed by acid pickling with a solution of 3% HF, 30% HNO_3_, and 67% H_2_O for 75 seconds before the tensile tests. Secondly, the surface of the sample was coated with an antioxidant of TO-12 glass protective lubricant supplied by Beijing Tianlichuang Company (Beijing, China) to prevent oxidation within hot deformation. Therefore, the oxidation state of the blank was neglected in this study. Uniaxial tensile tests were carried out on an INSTRON-5500R electronic universal material testing machine at 850 °C~1000 °C and 0.001 s^−1^~0.1 s^−1^. In order to obtain a uniform temperature distribution, the specimens were held at the preset temperature for 5 min before the tensile tests. In order to ensure the accuracy of the test results, each test was repeated three times. After the deformation, the sample was quenched with water immediately. Fixtures used in the tests to prevent the excessive deformation occurred at the clamping end of the thin plate samples which affect the accuracy of the test results during high temperature tensile tests at lower strain rates are shown in Figure 2b. Interrupted tensile tests at strains of 0.1, 0.3, 0.5, 0.7, and 0.9 were performed under different temperatures and strain rates to reveal the softening and hardening mechanisms of the TC31 titanium alloy within hot deformation.

The microstructures of the deformed specimens were characterized by Leica DMI3000M optical microscopy (OM) (Leica Microsystems Co. Ltd., Wetzlar, Germany), Quanta 200FEG field emission scanning electron microscopy (SEM) (FEI Systems Co. Ltd., Columbia, MD, USA), and electron backscatter diffraction (EBSD) (FEI Systems Co. Ltd.) respectively. The samples were taken from the center area (the red dotted box in Figure 2a). The samples were ground by 240#, 800#, 1200#, and 1500# metallographic sandpaper sequentially, and then electrically polished with a solution of 60% CH_3_OH, 34% C_4_H_10_O, and 6% HClO_4_ for 60 seconds. The samples used for OM and SEM also needed to be etched and the etching solution was composed of 13% HNO_3_, 7% HF, and 80% H_2_O. The EBSD characterization was performed at a step size of 0.3 μm and the test results were evaluated with OIM 6.1.4 software.

## 3. Results and Discussion

### 3.1. Dynamic Softening and Hardening Behavior

**Figure 3:**
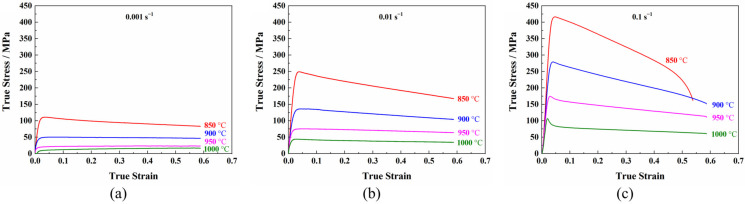
The flow behavior of the TC31 titanium alloy at different strain rates: (**a**) 0.001 s^−1^; (**b**) 0.01 s^−1^; (**c**) 0.1 s^−1^.

The true stress–true strain curves of the TC31 high-temperature titanium alloy under different deformation conditions are shown in Figure 3. It can be seen that the flow stress curves of the TC31 titanium alloy were similar when the deformation temperature was from 850 °C to 900 °C and the strain rate was from 0.001 s^−1^ to 0.1 s^−1^. At the initial stage of deformation, the flow stress increased rapidly to a peak stress, and then decreased with the increasing strain. The deformation can be rapidly transferred and diffused when the strain was low; therefore, no localized necking occurred [26]. Regarding the curve of 850 °C in Figure 3c, it is well known that the ductility decreases with the increase in the strain rate and the decrease in the deformation temperature [16,17]. When the TC31 titanium alloy was deformed at a lower temperature of 850 °C and a higher strain rate of 0.1 s^−1^, a fracture occurred before the true strain reached to 0.6; therefore, the curve is different to the rest of the curves. It should be noted that the other curves were interrupted at a strain of 0.6. When the deformation temperature increased from 850 °C to 900 °C, the flow stress decreased significantly with a maximum reduction of 54.5%.

The flow stress was almost steady with the increasing strain when the deformation temperature was from 950 °C to 1000 °C and the strain rate was from 0.001 s^−1^ to 0.01 s^−1^, indicating that the plastic deformation was relatively uniform under such conditions [27]. Therefore, it is suggested that the hot forming temperature of this alloy should be above 900 °C. When the deformation temperature was close to the β transition temperature which is 1025 °C for the TC31 titanium alloy, more α phase with a hexagonal close-packed structure would transform into the β phase with a body-centered cubic structure under lower strain rates condition [28]. The β phase has more slip systems and a higher stacking fault energy than the α phase, which contributes to a more uniform deformation.

The flow stress curves of the TC31 titanium alloy showed a discontinuous yielding phenomenon [29,30]. When the strain rate was 0.1 s^−1^, the flow stress reached a high peak value firstly at a lower strain condition, then the flow stress decreased rapidly. However, the flow stress decreased slowly with the increase in the strain after the lower yield point, showing the characteristics of continuous softening. The discontinuous yielding phenomenon became more and more obvious with the increase in the deformation temperature and strain rate [30,31], as shown in Figure 3c. One possible explanation for the stress drop was the stress-induced phase transformation (α→β) behavior which was observed during the hot deformation of the TC11 titanium alloy with α+β phase [30].

**Figure 4:**
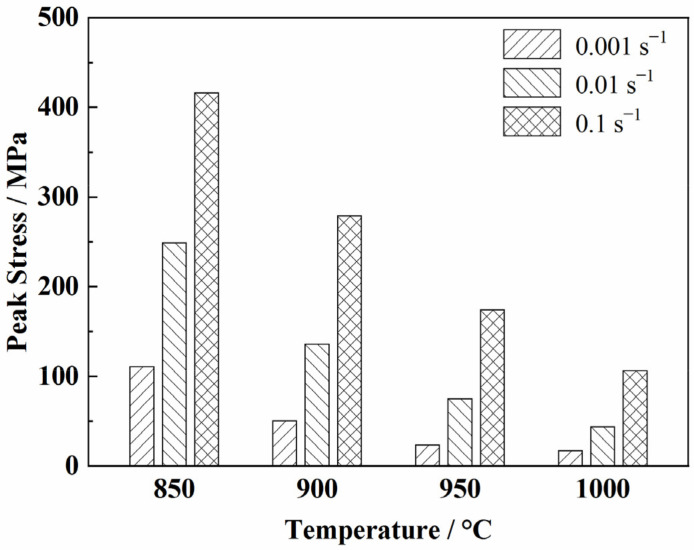
Peak stress at different temperatures and strain rates.

The peak stress of the TC31 titanium alloy increased with the increase in the strain rate and the decrease in the deformation temperature. When the strain rate was 0.1 s^−1^, the peak stress decreased sharply with the increase in the temperature. However, when the strain rate was 0.001 s^−1^, the values of the peak stress at 950 °C and 1000 °C were 23.2 MPa and 16.9 MPa, respectively. It can be inferred that the peak stress tended to be stable when the deformation temperature was higher than 950 °C at the strain rate of 0.001 s^−1^, as shown in Figure 4.

**Figure 5:**
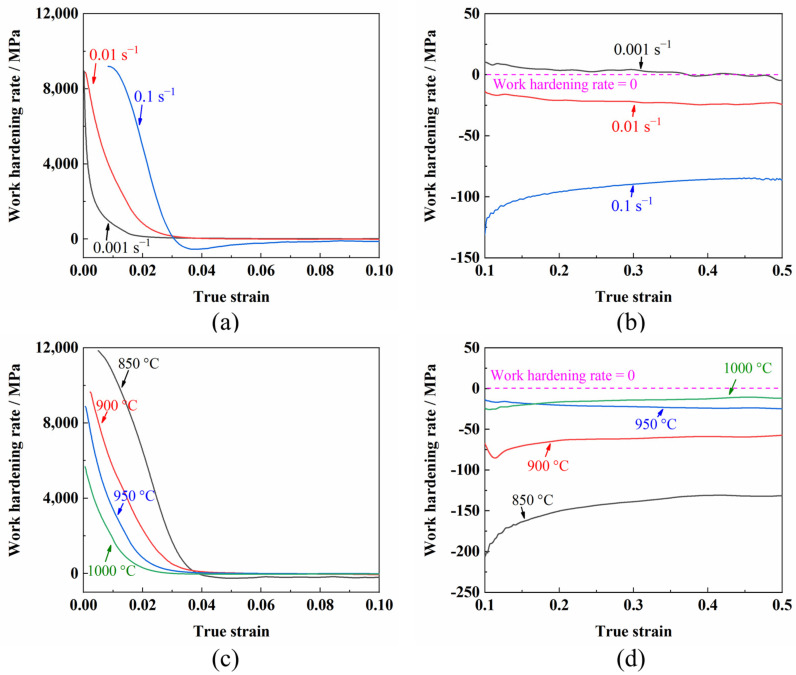
Work hardening rate at different deformation conditions: (**a**,**b**) 950 °C; (**c**,**d**) 0.01 s^−1^; ((**a**,**c**) are lower strain conditions; (**b**,**d**) are relatively higher strain conditions).

To further analyze the evolution of the flow stress during the hot deformation of the TC31 titanium alloy, the values of work hardening rates at a constant deformation temperature and strain rate were calculated from the true stress–true strain curves by the following formula [32]:

(1) $$\theta =\frac{\partial \sigma }{\partial \varepsilon }$$

where *σ* is the true stress and *ε* is the true strain. The variations between work hardening rate and hot deformation conditions are shown in Figure 5. The work hardening rate decreased rapidly with the increase in the strain at a lower strain condition (<0.05) as shown in Figure 5a,c, which can be attributed to the dynamic recovery of the TC31 titanium alloy [16,33]. Afterward, the variations in the work hardening rate were slight with the increase in the strain, as shown in Figure 5b,d. It can be seen from Figure 5a,b that the strain rate had a significant effect on the work hardening rate at 950 °C. The value of the work hardening rate was positive when the strain rate was 0.001 s^−1^ as shown in Figure 5b and the material was dynamically hardened. However, the work hardening rate changed from positive to negative when the strain rate increased from 0.001 s^−1^ to 0.1 s^−1^, demonstrating that the dynamic softening of the flow stress occurred during the deformation with higher strain rates. The dynamic softening phenomenon was more obvious when the strain rate was higher [17,27]. Particularly, the work hardening rate decreased firstly and then increased with the strain ranging from 0.03 to 0.06 and a strain rate of 0.1 s^−1^, which was attributed to the discontinuous yielding phenomenon [30,31]. The work hardening rate increased with the decrease in the deformation temperature at lower strain conditions as shown in Figure 5c, because much less recovery occurred at 850 °C than 1000 °C. However, with the increasing strain, the work hardening rate decreased with the decrease in the deformation temperature as shown in Figure 5d, which may be because of the grain growth with the increase in the deformation temperature [16,27].

**Figure 6:**
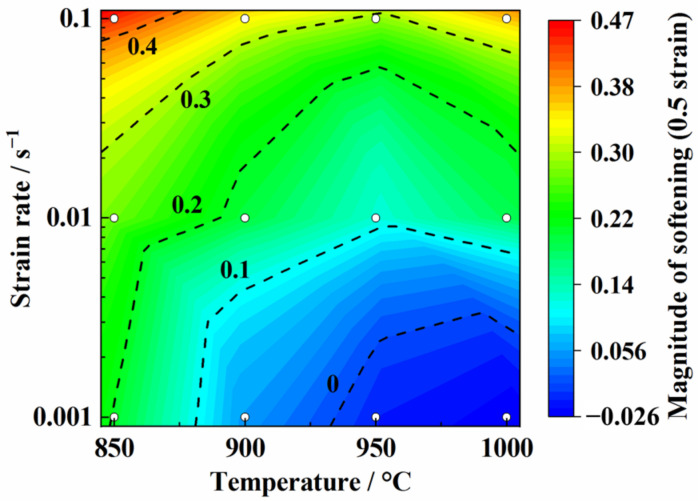
Flow softening coefficients at different temperatures and strain rates.

In order to quantitatively analyze the degree of dynamic softening during high temperature deformation, the flow softening coefficient (*M_s−_*_0.5_) was introduced and can be calculated from the following formulas [34,35]:

(2) $$M_{s-0.5}=\Delta \sigma /\sigma_{p}$$

(3) $$\Delta \sigma =\sigma_{p}-\sigma_{0.5}$$

where *σ_p_* is the peak stress and *σ*_0.5_ is the flow stress at the strain of 0.5. The hot deformation conditions had a significant effect on the flow softening coefficient of the TC31 titanium alloy as shown in Figure 6. The dynamic softening occurred in the region of higher strain rates (0.01 s^−1^ to 0.1 s^−1^) and the flow softening became more severe with the decrease in the deformation temperature. The values of the flow softening coefficients decreased with the decreasing strain rate and the increasing deformation temperature. Specifically, the values of the flow softening coefficients changed into negative values when the deformation temperature was 950 °C~1000 °C and the strain rate was 0.001 s^−1^, which indicated the occurrence of dynamic hardening, but the minimum value was only −0.026 and the degree of dynamic hardening was relatively slight.

Compared with aluminum alloys, the thermal conductivity of titanium alloys is considerably lower and the rise in temperature caused by deformation heat cannot be neglected. It is very challenging to accurately measure the value of the rise in temperature during the process of high temperature tensile deformation. Therefore, the prediction for the rise in temperature during the hot tensile deformation is very important and helpful to understand the flow behavior of titanium alloys. The amount of the temperature increment (Δ*T*) can be estimated from the following formulas [36,37,38]:

(4) $$\Delta T=\frac{p}{C}\int_{0}^{\varepsilon }\sigma d\varepsilon$$

(5) $$p=1+\frac{\dot{\varepsilon }}{3}$$

where *C* is the heat capacity coefficient and the value of the TC31 titanium alloy is about 4 N·mm^−2^·°C^−1^, *σ* is the true stress, *ε* is the true strain, and *p* is the thermal transfer coefficient and is related to the strain rate. It can be concluded that the Δ*T* at 850 °C and 900 °C was much greater than that at 950 °C and 1000 °C because the flow stress decreased rapidly with the increase in the deformation temperature, particularly under the higher strain rate conditions. Therefore, the deformation heat will intensify the dynamic softening of the titanium alloy under the lower temperature conditions [35,38].

### 3.2. Micro Mechanisms of Dynamic Softening and Hardening

Micro variables such as grain size, dislocation density, grain boundary, recrystallization, phase volume fraction, phase morphology, and voids in the titanium alloy are closely related to the high temperature deformation conditions which include the deformation temperature, strain rate, and strain [16,32,38,39]. The evolution of those micro variables will determine the dynamic softening and hardening of the flow stress curves. To fully reveal the micro mechanisms of dynamic softening and hardening during the deformation, careful microstructure characterization and analysis are necessary.

#### 3.2.1. The Effect of Deformation Temperature

**Figure 7:**
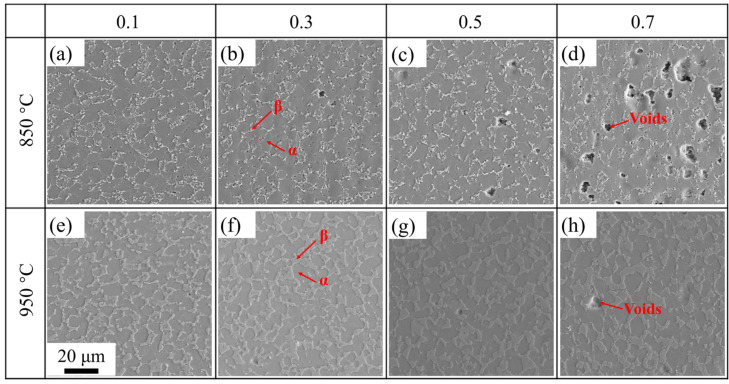
Microstructure at different temperatures and strains when deformed at 0.01 s^−1^: (**a**–**d**) 850 °C; (**e**–**h**) 950 °C; (**a**,**e**) strain 0.1; (**b**,**f**) strain 0.3; (**c**,**g**) strain 0.5; (**d**,**h**) strain 0.7.

The deformation temperature had a significant effect on the flow stress of the TC31 titanium alloy during high temperature deformation. The TC31 titanium alloy showed an obvious softening phenomenon when it was deformed at 850 °C, but the softening phenomenon gradually weakened with the increase in the deformation temperature. When the deformation temperature was from 950 °C to 1000 °C and the strain rate was 0.001 s^−1^, slight dynamic hardening occurred during the deformation process. Figure 7 shows the evolution of the microstructure with increasing strain at 850 °C and 950 °C at a strain rate of 0.01 s^−1^. It is well known that the β phase with a body-centered cubic structure has more slip systems than the α phase with a hexagonal close-packed structure; therefore, the deformation of the β phase is easier than the α phase and the β phase can withstand greater plastic deformation [20,40]. In the process of plastic deformation, the amount of the deformation for the α phase and the β phase is different, and the stress concentration will occur at the interface between the α phase and the β phase [1,18,28]. When the deformation temperature was 850 °C, the volume fraction and morphology of the β phase had fewer differences compared with the initial microstructure and the relatively large stress concentration that occurred at the phase boundaries with the increase in the strain. The volume fraction of the voids was about 11.2% when the true strain was 0.7. The appearance and growth of voids led to the rapid reduction in the flow stress and the softening of the TC31 titanium alloy, which was more obvious under lower temperature conditions [26].

When the deformation temperature increased from 850 °C to 950 °C, the volume fraction of the β phase increased from 20% to 41% at the strain of 0.7 and the size of the β phase also had a significant coarsening. It is well known that the sliding resistance of the α/β phase interface is lower than that of the α/α interface and the β/β interface [40,41]. Therefore, the increase in the proportion of the α/β phase interface which is attributed to the rise of the volume fraction of β phase is beneficial to grain boundary sliding, and the deformation consistency is also improved simultaneously [22,24,41]. At the same time, the recovery and recrystallization at 950 °C was more efficient than those at 850 °C, which led to a lower dislocation density near the phase and grain boundaries [18,39,40]. Hence, the volume fraction of the voids was less than 1% when the deformation temperature was 950 °C and the strain was 0.7, and the flow stress was almost steady during the deformation, as shown in Figure 3b.

#### 3.2.2. The Effect of the Strain Rate

When the TC31 titanium alloy was deformed at 950 °C, dynamic hardening occurred at a strain rate of 0.001 s^−1^ and dynamic softening occurred at the strain rates from 0.01 s^−1^ to 0.1 s^−1^. The dynamic softening was more obvious with the higher strain rate, indicating that the strain rate had a significant effect on the evolution of the microstructure.

**Figure 8:**
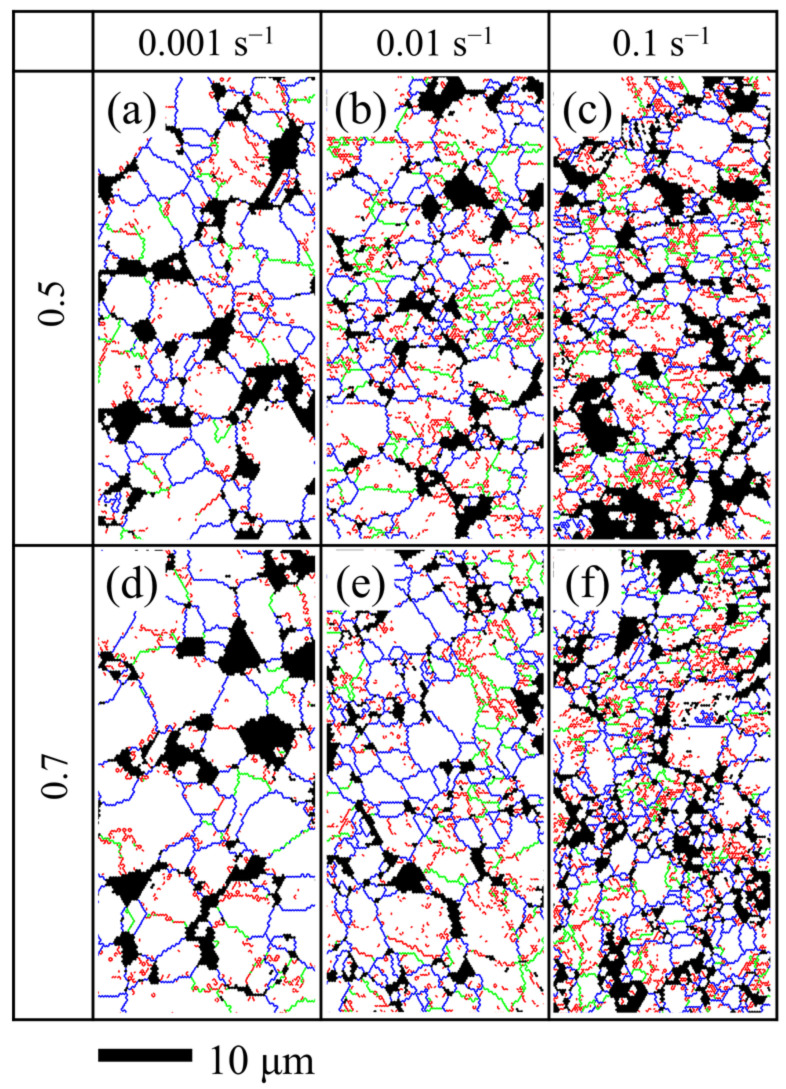
The distributions of the grain boundary at different strain and strain rates when deformed at 950 °C: (**a**–**c**) strain 0.5; (**d**–**f**) strain 0.7; (**a**,**d**) strain rate 0.001 s^−1^; (**b**,**e**) strain rate 0.01 s^−1^; (**c**,**f**) strain rate 0.1 s^−1^ (red lines represent LAGBs less than 5°, green lines represent MAGBs between 5°~15°, blue lines represent HAGBs more than 15°).

Figure 8 shows the distribution of the grain boundary at different strain rates, where the blue, green, and red lines represent high-angle grain boundaries (HAGBs), medium-angle grain boundaries (MAGBs), and low-angle grain boundaries (LAGBs), respectively. When it was deformed at the strain rate of 0.001 s^−1^, the fraction of LAGBs decreased rapidly and the average grain size increased significantly, as shown in Figure 8a,d. Compared with the initial grain size of 2.80 μm, the grain sizes at the strain of 0.5 and 0.7 increased to 3.08 μm and 3.66 μm, respectively. When it was deformed with the strain rate of 0.1 s^−1^, the fraction of LAGBs decreased from 61.8% to 49.3% at the strain of 0.5 and the grains were also refined compared with the initial microstructure, as shown in Figure 8c. The average grain sizes at the strain of 0.5 and 0.7 were 2.19 μm and 1.87 μm, respectively. When the strain rate was 0.01 s^−1^, the refinement was slight and the average grain sizes were 2.52 μm and 2.39 μm at the strain of 0.5 and 0.7, respectively.

It can be summarized that during the deformation at 950 °C, the average grain size decreased with the increase in the strain rate at the strain of 0.5 or 0.7. At the lower strain rate of 0.001 s^−1^, the grains appeared almost equiaxed with a few sub-grains and the sub-grains reduced rapidly compared with the initial microstructure [20]. The obvious reduction of the sub-grains could be attributed to DRX [42]. With the progress of DRX, the migration of HAGBs could consume the dislocation and reduce the sub-grains [36,39]. Fully DRX was completed before the strain reached 0.5 under 0.001 s^−1^ condition and then grain growth occurred with the increase in the strain. It should be noted that the recrystallized grains grew up significantly under the strain rate of 0.001 s^−1^; therefore, the softening effect caused by DRX decreased with the decreasing strain rate and the flow stress exhibited dynamic hardening. Similar dynamic hardening was also reported during the superplastic deformation of the TC4 titanium alloy [24,34]. It can be seen from Figure 8a–f that the amount of the recrystallized grains without LAGBs inside increased and the average grain size decreased with the increasing strain rate. At a higher strain rate of 0.1 s^−1^, the deformation inhomogeneity became more obvious. The deformation energy was higher around the grain boundaries than that at other sites [11,28]; therefore, most of the recrystallized grains nucleated near the grain boundaries. The deformation time of the sample deformed at a strain rate of 0.1 s^−1^ was too short for the growth of new recrystallized grains [24]. Therefore, the flow stress exhibited dynamic softening under the higher strain rate conditions.

**Figure 9:**
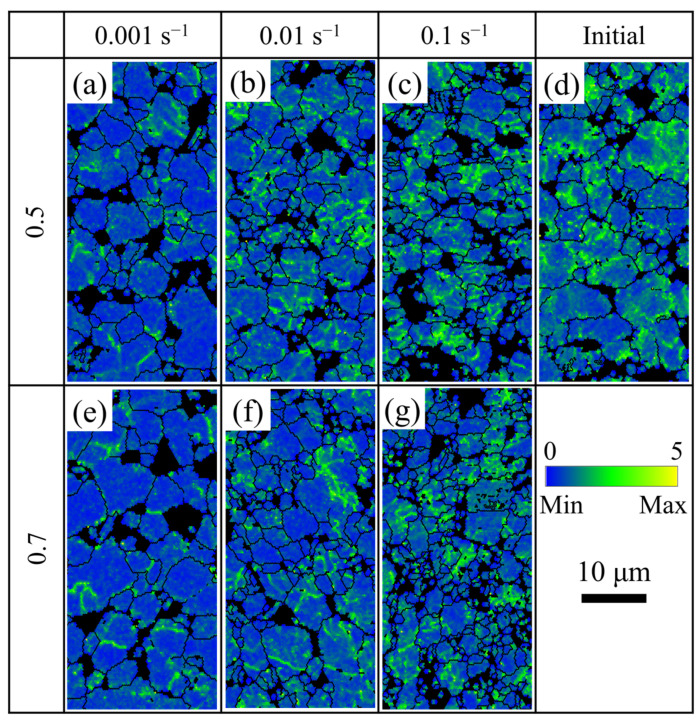
The distributions of KAM at different strains and strain rates when deformed at 950 °C: (**a**–**c**) strain 0.5; (**d**) initial; (**e**–**g**) strain 0.7; (**a**,**e**) strain rate 0.001 s^−1^; (**b**,**f**) strain rate 0.01 s^−1^; (**c**,**g**) strain rate 0.1 s^−1^.

The high-density dislocations around the grain boundaries could provide favorable sites for the nucleation of recrystallization [19,28]. In order to further analyze the effect of the strain rates on the deformation mechanism of the TC31 titanium alloy, the Kernel Average Misorientation (KAM) maps under different deformation conditions are shown in Figure 9. When the strain rate was 0.001 s^−1^, the LAGBs had enough time to evolve into HAGBs leading to the decrease in the dislocation density. The mean value of KAM decreased rapidly from 1.069 at the initial stage to 0.735 at the strain of 0.5. When the strain was further increased to 0.7, the mean value of KAM was 0.716 and the reduction was not significant. It can be seen from Figure 9a,e that the dislocation density was at a lower level with a strain rate of 0.001 s^−1^ and the strain of 0.5 and 0.7, which would hinder the nucleation of dynamic recrystallization [24]. Therefore, the main deformation mechanism may have been grain boundary sliding and the grain size increased gradually [20,24]. The mean value of KAM decreased slightly with the increase in the strain when the strain rate was 0.01 s^−1^. The mean values of KAM were 0.917 and 0.824 at the strain of 0.5 and 0.7, respectively. When the strain rate was 0.1 s^−1^, only a few seconds were needed for the strain to reach to 0.5, and the mean values of KAM at the strain of 0.5 and 0.7 were 0.975 and 0.958, respectively. It can be seen from Figure 9c,d,g that the dislocation density remained almost steady with the increasing strain under the higher strain rate condition of 0.1 s^−1^; therefore, the nucleation sites for dynamic recrystallization were abundant [28] and the dynamic softening of the TC31 titanium alloy was obvious.

#### 3.2.3. The Effect of Strain

**Figure 10:**
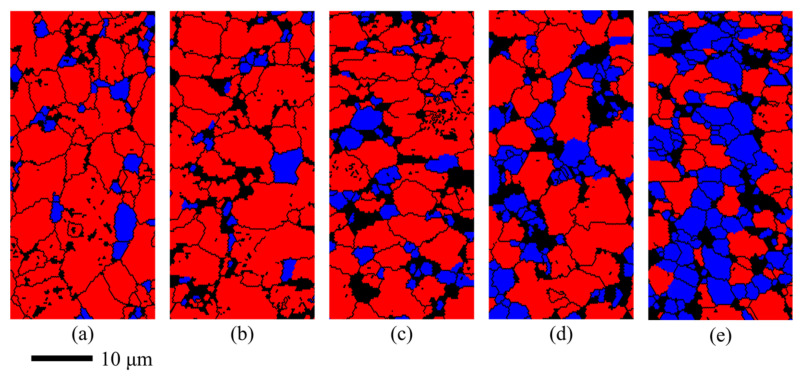
The distributions of recrystallization at different strains when deformed at 950 °C and 0.01 s^−1^: (**a**) Initial; (**b**) 0; (**c**) 0.1; (**d**) 0.5; (**e**) 0.9 (blue represents the recrystallized grains, red represents the deformed grains).

**Figure 11:**
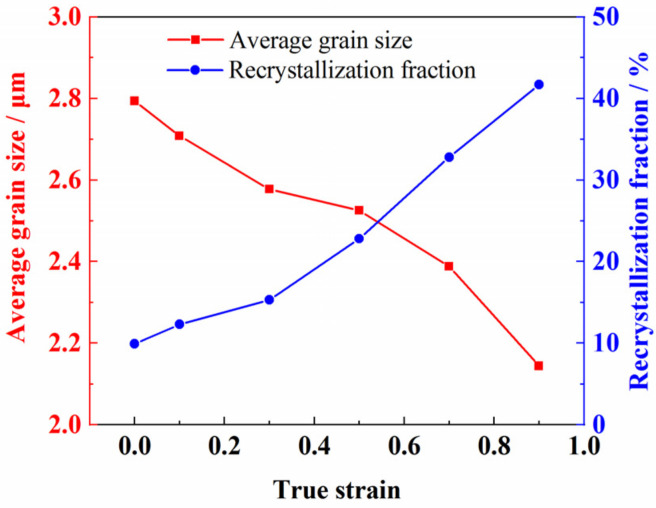
The relationships between the recrystallization fraction, the average grain size, and the true strain when deformed at 950 °C and 0.01 s^−1^.

The flow softening coefficient was only 0.12 when the deformation temperature was 950 °C and the strain rate was 0.01 s^−1^, indicating that the dynamic softening phenomenon caused by recrystallization was very slight [8]. Figure 10 shows the evolution of recrystallization at different strains, where the blue area is the recrystallized grain. It can be seen from Figure 10a that the volume fraction of recrystallization of the as-received sheet was about 8.1%, indicating that the as-received sheet had not been annealed sufficiently. The volume fraction of recrystallization increased to 9.5% after the sample was soaked at 950 °C for 5 min as shown in Figure 10b. It can be concluded that the effect of static recrystallization was limited because of the short holding time [1]. The volume fractions of recrystallization were 12.3%, 22.8%, and 41.7% at the strain of 0.1, 0.5, and 0.9, respectively. The results show that the degree of recrystallization increased rapidly with the increase in strain when the strain rate is 0.01 s^−1^. Because the strain rate was relatively higher, and the time during the deformation process at 950 °C was relatively shorter, the grain sizes decreased from 2.80 μm at the initial stage to 2.13 μm at the strain of 0.9 gradually. The relationships between the recrystallization fraction, the grain size, and the true strain are shown in Figure 11.

**Figure 12:**
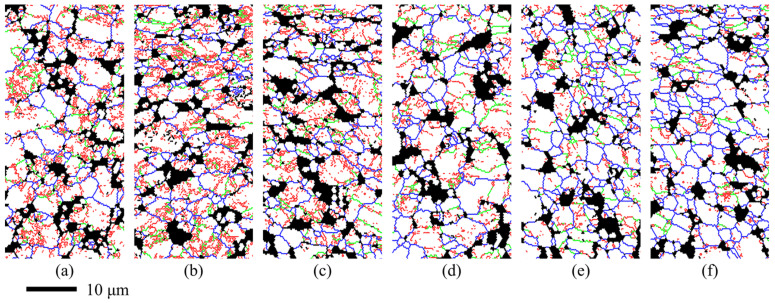
The distributions of the grain boundary at different strains when deformed at 950 °C and 0.01 s^−1^: (**a**) Initial; (**b**) 0.1; (**c**) 0.3; (**d**) 0.5; (**e**) 0.7; (**f**) 0.9 (red lines represent LAGBs less than 5°, green lines represent MAGBs between 5°~15°, blue lines represent HAGBs more than 15°).

Figure 12 shows the distribution of the grain boundaries and the subgrain boundaries at different strains when the deformation temperature was 950 °C and the strain rate was 0.01 s^−1^. A high density of subgrain boundaries including LAGBs and MAGBs with a total proportion of 75.6% was observed at the initial stage as shown in Figure 12a and the proportion decreased sharply to 44.9% at the strain of 0.9 as shown in Figure 12f. When the strain was lower, the distribution of the subgrain boundaries was not uniform and the subgrain boundaries migrated from the inside of the grain to the grain boundaries [20]. During the hot deformation process, the proportion of the subgrain boundaries decreased and the proportion of HAGBs increased gradually accompanied with some new fine grains formed around the grain boundaries [20,35]. The amount of new fine grains increased significantly when the strains were 0.7 and 0.9, indicating that the recrystallization was active.

**Figure 13:**
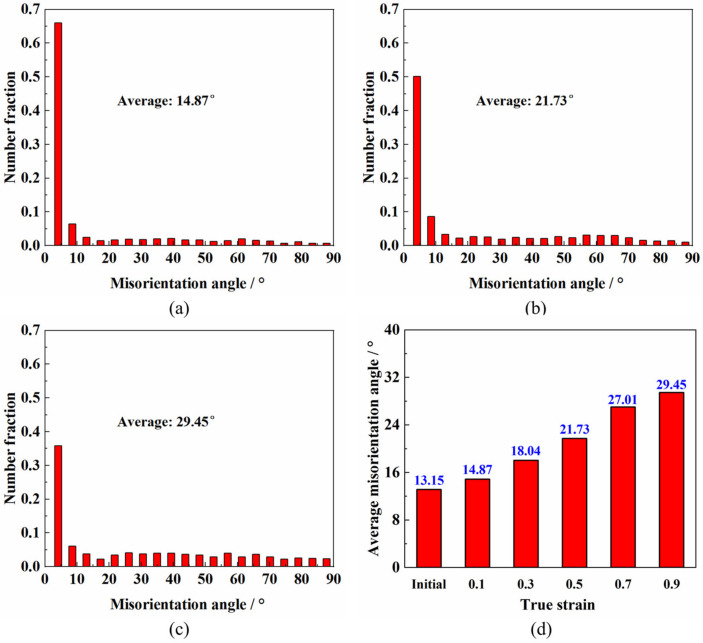
The distributions of the misorientation angle at different strains when deformed at 950 °C and 0.01 s^−1^: (**a**) 0.1; (**b**) 0.5; (**c**) 0.9; (**d**) the evolution of the average misorientation angle with strain.

Figure 13 shows the distribution of misorientation at different strains. It can be seen from Figure 13a–c that the proportion of HAGBs was relatively lower and the distribution was uniform, but the proportion of LAGBs was very high [20,28]. The average misorientation increased approximately linearly with the increase in the strain, which was 14.87° and 29.45° at the strain of 0.1 and 0.9, respectively. The increase in the average misorientation was related to the dynamic recrystallization during hot deformation [39].

**Figure 14:**
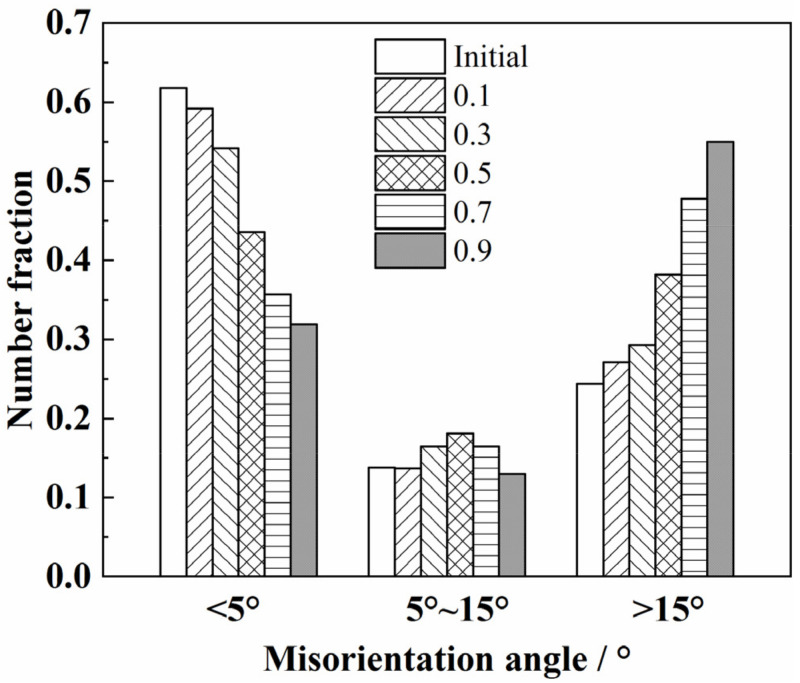
The evolution of the misorientation angle at different strains when deformed at 950 °C and 0.01 s^−1^.

The variations in the fractions of LAGBs, MAGBs, and HAGBs during the hot tensile tests are summarized in Figure 14. The proportion of HAGBs increased slowly when the strain was less than 0.3 and increased rapidly when the strain was greater than 0.3. The proportion of LAGBs decreased gradually. However, the variation of MAGBs was not monotonous. The proportion of MAGBs increased gradually when the strain was less than 0.5 and decreased gradually when the strain was greater than 0.5. Dynamic recrystallization mechanisms include continuous dynamic recrystallization (CDRX) characterized by the rotation of the subgrain boundary and discontinuous dynamic recrystallization (DDRX) characterized by the nucleation and growth of grains with small sizes [40,43,44]. The MAGBs is the transitional stage of evolution from LAGBs to HAGBs; therefore, the variation of MAGBs is closely related to the types of recrystallization [20,39]. During the process of CDRX, the proportion of MAGBs increases with the increase in strain, but the proportion of MAGBs decreases with the increase in strain when DDRX occurred [39]. During the hot deformation of the Ti55 high-temperature titanium alloy, DDRX became the main mechanism of dynamic softening with the increase in the strain [20]. For the TC31 titanium alloy deformed at 950 °C and 0.01 s^−^^1^, the CDRX was the dominant factor of dynamic softening when the strain was less than 0.5. With the increase in the strain, the effect of DDRX enhanced gradually and the DDRX became the dominant factor of dynamic softening when the strain was greater than 0.5.

**Figure 15:**
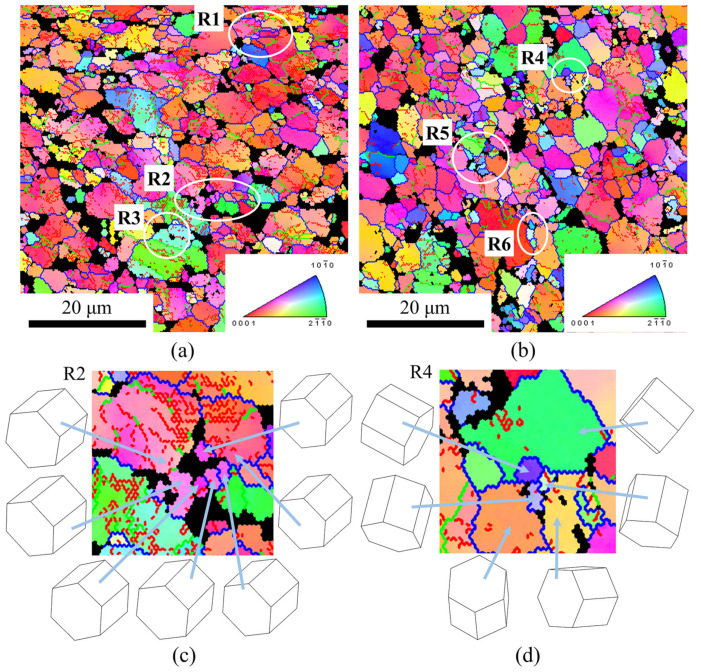
The IPF at different strains when deformed at 950 °C and 0.01 s^−1^: (**a**,**c**) 0.3; (**b**,**d**) 0.7.

Figure 15 shows the IPF at different strains. When the strain was 0.3, it can be seen from Figure 15a that a few recrystallized grains with the grain size of about 1 μm appeared at the boundary of coarse grains, and their orientations were close to that of the adjacent coarse grains, as shown in Figure 15c. There were a large number of subgrain boundaries formed by the accumulation of dislocations inside the coarse grains. It was indicated that the CDRX occurred [21,39]. It can be seen from Figure 15b that the amount of the recrystallized grains increased significantly with the increase in the strain to 0.7, and the recrystallized grains were distributed in clusters. Their orientations were significantly different from that of the adjacent un-recrystallized coarse grains as shown in Figure 15d, which is considered to be a typical feature of DDRX [21,39]. The amount of the subgrain boundaries within the un-recrystallized grains reduced greatly and the grain boundaries of the un-recrystallized grains were serrated, demonstrating that the mechanism was DDRX [20,21].

**Figure 16:**
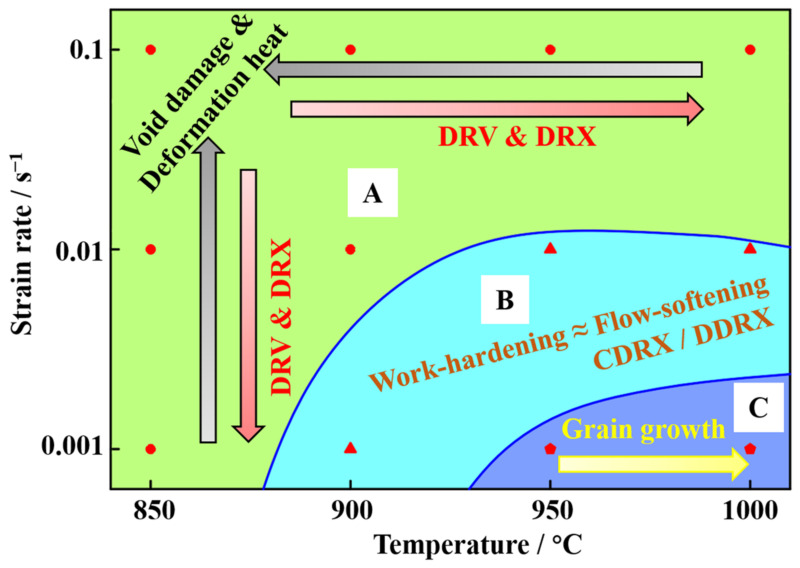
The hot deformation behaviors and the microstructure evolution mechanisms of the TC31 titanium alloy.

It is well known that the hot deformation parameters have a significant influence on the hot deformation behavior and the evolution of the microstructure [16,17,20]. Qiu et al. [27] pointed out that the flow softening of the SP700 titanium alloy was mainly attributed to DRX during the hot deformation with relatively low temperatures and relatively high strain rates, but the steady-state flow characteristics appeared with the increasing temperature and the decreasing strain rate. During the superplastic deformation of the TC4 titanium alloy, the flow stress increased with the increase in strain during the deformation with a strain rate of 0.0001 s^−1^ and a temperature ranging from 850 °C to 950 °C [24]. The dynamic hardening rate increased with the increasing deformation temperature due to the more obvious grain growth during the deformation [24]. The deformation behavior and microstructure evolution of the TC31 titanium alloy were similar with the aforementioned SP700 and the TC4 titanium alloy. In the future forming of the TC31 titanium alloy component, one should carefully choose the parameters such as temperature and strain rate to control the forming process and post-form properties according to the relationships between the parameters, the microstructure, and the flow stress. Based on the above analysis, the dynamic softening and hardening behaviors of the TC31 titanium alloy are summarized in Figure 16. In the upper left zone A, with a relatively lower temperature and a higher strain rate, the void damage and the deformation heat generation were the important reasons that caused the dynamic softening of the flow stress with the increase in the strain [37]. The softening effect of DRV and DRX increased with the increasing temperature and decreasing strain rate [18,23,27]. When the deformation temperature was higher than 950 °C and the strain rate was lower than 0.001 s^−1^, such as in zone C in Figure 16, the dynamic hardening characteristics of the flow stress curves were mainly attributed to the grain growth [11,24]. In zone B, the effect of work hardening caused by dislocation accumulation and grain growth was approximately equal to that of the flow softening caused by DRV, DRX, or void damage [8,28]. In zone B, the recrystallization mechanism was mainly CDRX at the initial deformation stage, and transformed into DDRX with the increase in strain. Based on the above analysis, it is suggested that TC31 titanium alloy components are formed within zone B to obtain a relatively uniform deformation and good post-form properties.

## 4. Conclusions

The dynamic softening and hardening behavior and microstructure evolution of the TC31 titanium alloy during high temperature tensile deformation have been systematically investigated in this paper. Based on the analysis of the experimental results, the main conclusions are summarized as follows:

1. The TC31 titanium alloy exhibited obvious softening behavior during hot tensile deformation at a temperature of 850 °C and a strain rate of 0.001 s^−1^~0.1 s^−1^; with an increase in the deformation temperature to 950 °C~1000 °C and an increase in the strain rate to 0.1 s^−1^, discontinuous yielding occurred; quasi-steady flow appeared at a temperature of 950 °C~1000 °C and a strain rate of 0.01 s^−1^; with a decease in the strain rate to 0.001 s^−1^, slight dynamic hardening phenomenon occurred. Therefore, a careful selection of the forming temperature and the strain rate of the TC31 titanium alloy sheet is very important to control the dynamic softening or dynamic hardening during high temperature deformation.
2. When the deformation temperature increased from 850 °C to 950 °C, the volume fraction of the β phase increased from 20% to 41% after it deformed to a strain of 0.7 with a strain rate of 0.01 s^−1^, whereas the volume fraction of voids was significantly reduced from 11.2% to less than 1%. The increased fraction of the β phase at higher temperatures improved the deformation compatibility and reduced the void damage. Therefore, a relatively high deformation temperature is recommended for the forming of complex TC31 titanium alloy components to avoid the void damage.
3. When the TC31 titanium alloy was deformed at 950 °C, the grains grew up at the strain rate of 0.001 s^−1^ and were refined at the strain rate from 0.01 s^−1^ to 0.1 s^−1^, and the refinement was more significant under the higher strain rate conditions. The appropriate strain rate should be about 0.01 s^−1^ during the forming of the TC31 titanium alloy sheet considering both the grain coarsening and uniform deformation.
4. When the samples were deformed at a temperature of 950 °C and a strain rate of 0.01 s^−1^, the proportion of MAGBs firstly increased when the strain was less than 0.5 and then decreased gradually when the strain was greater than 0.5. The main recrystallization mechanism transformed from CDRX to DDRX and the grain sizes decreased gradually with the increase in the strain.

## Figures and Tables

**Table 1:** Chemical composition of the TC31 titanium alloy.

Element	Al	Sn	Zr	Nb	Mo	W	Si	Ti
wt.%	5.21	3.45	3.47	1.26	1.24	0.39	0.20	Bal.

## Data Availability

Not applicable.
